# Multiple Pseudo-Plastic Appearance of the Dynamic Fracture in Quasi-Brittle Materials

**DOI:** 10.3390/ma13214976

**Published:** 2020-11-05

**Authors:** Gianmario Riganti, Ezio Cadoni

**Affiliations:** DynaMat Laboratory, University of Applied Sciences and Arts of Southern Switzerland, 6952 Canobbio, Switzerland; gianmario.riganti@supsi.ch

**Keywords:** quasi-brittle material, DAMP model, damage propagation, material model, split Hopkinson bar, numerical simulation

## Abstract

Understanding and simulating the dynamic response of quasi-brittle materials still remains as one of the most challenging issues in structural engineering. This paper presents the damage propagation material model (DAMP) developed in order to obtain reliable results for use in structural engineering practice. A brief overview focuses on the differences between fracture mechanics studies, and engineering material modelling is presented to highlight possible guideline improvements. An experimental dynamic test performed on ultra-high-performance concrete specimens was used to obtain evidence of the physical behaviour of brittle materials with respect to specimen size variations and, consequently, to verify the reliability of the material equations proposed. Two widely used material models (RHT and M72R3), as representatives of the classical brittle material models for structural purposes, and the proposed material model are compared. Here, we show how: (i) the multiple structural strength of brittle materials arises from the damage propagation process, (ii) there is no contradiction between fracture mechanics and the engineering approach once the velocity of damage propagation is chosen as fundamental material property and (iii) classical dynamic material models are intrinsically not objective with related loss of predictive capability. Finally, the original material model equation and the experimental strategy, dedicated to its extended verification, will be shown in order to increase the design predictiveness in the dynamic range considering structure and specimen size variations. The dynamic stress increasing factor (DIF), experimentally observed and widely recognised in literature as a fundamental concept for quasi-brittle material modelling, has been reviewed and decomposed in its geometrical and material dependencies. The new material model defines its DIF starting from the physical quantities of the damage propagation velocity applied to the test case boundary conditions. The resultant material model predictiveness results improved greatly, demonstrating its ability to model several dynamic events considering size and dynamic load variations with a unique material property set without showing contradictions between numerical and experimental approaches.

## 1. Introduction

Currently, in the design and construction of critical infrastructures (i.e., power plants, nuclear reactors, protective structures, dams, bridges), there is a great and renewed interest in understanding the mechanical response of quasi-brittle material and structural components subjected to high dynamic loading including earthquakes, man-made or natural hazards, oil perforations, etc. Current structural design is principally based on the knowledge of the material failure limits and on the capability of the material model to represent the actual behaviour of both material and structure. Despite the extensive experimental and theoretical investigations developed in the last several decades, structural design applied to quasi-brittle materials has brought about several open questions regarding the reliability and the predictive capability of the material models used. Better integration between the material model equations and the real physical behaviour of materials in a wider strain rate range is expected.

The quasi-brittle material failure is characterised by a crack propagation process in which the final reduction of the structural element resistance is determined by the loss of its integrity. The process of brittle fracture has been investigated in different ways based on researchers’ scientific fields and practical interests. It is possible to distinguish different approaches, e.g., based on: (i) fracture mechanics which develops theoretical models to describe crack propagation; (ii) structural engineering use (the purpose is to apply structural calculation methods to produce structural outputs); (iii) experimental dynamic with the goal of collecting raw data representative of the material behaviour. The first two approaches use different equations and theories to treat experimental data.

The basic idea of fracture mechanics highlights how the presence of a dominant crack into a continuum body creates a perturbation into the stress field, leading to the stability or instability of the original crack eventually evolving then to structure failure. In the case of ideal elastic material relation, the crack geometry acts as the dominant parameter governing the fracture stability and then severely conditioning the admissible loads. Within this framework, the stress intensity factor is the parameter allowing to compute the new resistance limit of a cracked body. The stress intensity factor is highly geometry-dependent [1] and it is widely used in linear elastic structural analysis, e.g., in weld resistance prediction for metallic structures.

The original Griffith’s idea described above was upgraded and current fracture dynamics considers the effect of a wide spread of interacting crack flaws [2], lately resulting in specimen failure. In this case, the effect of the spread of cracks into the material is identical to that of a dominant crack. Thus, micro-scale information plays a dominant role in fracture mechanics [3]. Instead, engineering models of quasi-brittle materials do not consider any interaction between geometrical parameters and failure loads. This is in contrast with the physical presence of a large number of cracks within the quasi-brittle material wherein each one interacts with the global structural resistance as shown in the single crack case. The engineering approach to quasi-brittle materials is focused on the macroscopic material response looking for the threshold value of the apparent failure stress governing the specimen or structure resistance. In practical engineering approaches, the failure limit is dependent uniquely on material properties and failure mode, while it is independent from the size of the structure or from the geometry of the specimen. In order to overcome the intrinsic limitations of the described approaches, a new material model was developed. The purpose of this paper is to present the damage propagation material model (hereafter referred to as DAMP) and to assess its suitability to describe the dynamic fracture of quasi-brittle materials by comparing it with the results obtained with two widely used models (M72R3 [4] and RHT [5,6]).

The DAMP model has been developed in the frame of a research project aiming to investigate the dynamic response of retrofitting slabs combining different layers of reinforced concrete and UHP(FR)C. The project was organised in different phases: first, experimental blasting tests on slabs with different contact and stand-off charges were performed, followed by experimental dynamic tests over specimens, varying their size, notch geometries, dynamic loading conditions and failure mode. Finally, data analysis, material modelling, simulation of experimental tests and structural assessment were developed.

A large set of dynamic experimental test data was collected and a general approach to the dynamic structural analysis based on a new material constitutive equation was implemented. Structural calculation reliability was put in relation with material constitutive equations showing how the numerically calculated structural failure loads can be strongly different from the experimental one when a single dynamic test is accurately reconstructed. More than 400 dynamic tests were performed considering several stress states and using special shaped specimens and results were used to validate the new material model.

By focusing mainly on material models, we wish to answer in this paper the basic question of whether the classical material model equations are suitable to represent the quasi-brittle material model response, or whether the DIF (structural strength) concept should be reconsidered as a structural evidence rather than a material fundamental property. To do that, a benchmark was considered, and the response obtained by a classical material model represented here was compared. It is evident how a greater applicability and accuracy were reached by modifying the constitutive equation.

## 2. Outlook of the Quasi-Brittle Materials Modelling in Dynamics

During a blast event, a structure is subjected to high variations in loading. At each point, the local stress intensity and load duration can vary by several order of magnitudes with respect to those at another point. Variations are due to the wave propagation inside the structure, to the type of loading wave and to the geometrical position considered. In order to be reliable, the material model equation used to calculate the structural response should be able to reproduce the real material behaviour in a wide range of dynamic pulses; otherwise, the material model reliability will be insufficient. Indeed, the material model parameters should be the same ones identified by different dynamic tests. A two-sided perspective, known as the principle of material objectivity [7], should always be satisfied to ensure reliable engineering analysis. Despite this clear general material principle, the application of a priori reliable calculation assessment is far from being obtained for a generic structure made of quasi-brittle materials.

Many material models were created for structural engineering purposes. For the sake of brevity, we introduce only the two most commonly used models: the M72R3 and the RHT, which is widely used in dynamic applications dedicated to blast, perforation and damage calculation [8,9,10]. The material model class to which the two cited models belong is characterised by the following baseline: (i) the material failure stress is defined by the stress tensor components and by a damage function reproducing stress softening; (ii) the volume of material instantaneously goes in a plastic regime, accumulating damage driven by stress and strain; (iii) a stress dynamic increasing factor (DIF) or rate sensitivity function is adopted to describe the material failure and to adapt the maximum stresses to the dynamic conditions [11]. The great advantage of those models consists in their suitability for automatic structural analysis, including linear and nonlinear cases as well as fluid–structure interaction. Their main limitations are the great difficulties faced to find unique and satisfying material parameters allowing the user to obtain reliable solutions for arbitrary shapes even when those parameters are obtained by fitting real test data [8,12,13,14,15,16]. This difficulty vanishes the great value of systematic applicability of engineering criteria, generating uncertainty over the design requirements and the related failure risks and costs. To overcome these issues, several authors promoted foster experimental investigations, considering the split Hopkinson Bar test over a wide specimen scale [17,18,19], or over sub-structures or real components such as the cantilever beam and other structural tests [20,21]. Although many attempts have been made, the improvement of the reliability of numerical simulation for structures made of quasi-brittle material subjected to high dynamic loading is still a challenging and promising topic [22,23,24].

### Classical Concrete Material Model

Classical material models used for quasi-brittle materials are based on the following hypotheses: (i) the material homogenisation at the characteristic scale and (ii) the representativeness of the test conditions with respect to the final event. These hypotheses allow to apply to concrete both of the dynamic equations developed for homogeneous materials and the continuity Equation (1), which links the specimen displacement *u* to the material strain tensor $\underline{\epsilon }$ under the assumption of a strain uniformly distributed at the interior of the volume of control. In case of the split Hopkinson bar (SHB) test, the continuity equation leads to Equation (2), obtained by an analytical solution. The specimen average strain $\epsilon_{sp.}$ is then obtained by the specimen length $L_{sp.}$ and the stress wave propagation speed $C_{0}$ multiplied by the integral of the reflected strain $\epsilon_{r}\left(t\right)$ which is experimentally recorded. When the material is calibrated by numerical reconstruction of the SHB test, Equation (1) is used by the FEM solver applying the material models. The material model and its failure surface Equation (3) allows to obtain the failure stress $\sigma_{y}$ as a function of the stress tensor $\underline{\sigma }$, and of the compressive and tensile strength $f_{c}$ and $f_{t}$. After having reached the failure stress, the material accumulates damage according to the function of the total plastic strain $\epsilon_{p}$ accumulation Equation (4). The plastic strain is the scalar parameter used to compute the damage parameter *D* Equation (5), which in turn allows to model the softening by the damaged stress $\sigma_{d}$ Equation (6). The damaged stress is then dynamically scaled using a strain rate factor *R* calculated by the time variation of the total plastic strain and the damage Equation (7):

(1) $$\underline{\epsilon }=f\left(\underline{u}\right)$$

(2) $${\epsilon_{sp.}\left(t\right)}_{eng.}=-\frac{2\cdot C_{0}}{L_{sp.}}\int_{0}^{t}\epsilon_{R}\left(t\right)\;dt$$

(3) $$F(\sigma ,f_{c},f_{t},\epsilon )=0$$

(4) $$\epsilon_{p}=e\left(\underline{\epsilon }\right)$$

(5) $$D=D(\delta \epsilon_{p},\epsilon_{fail})$$

(6) $$\sigma_{d}=\sigma_{d}\left(\sigma_{y},D\right)$$

(7) $$R=R\left(\frac{d\epsilon_{p}}{dt},D\right)$$

The set of equations (Equations (1)–(7)) represent the conceptual framework used by the damage plasticity material class. For example, RHT [5], Holmquist [25], AFC [26] and M72R3 [4] models use the presented schematisation.

Up to now, the vast majority of the experimental efforts addressed to properly understand the quasi-brittle materials’ dynamic responses (tension, compression, shear) highlighted the existence of an increase of the material failure limit. For example, for concrete, several expressions of DIF were proposed, e.g., in fib Model Code [27]. This method is the synthesis of the average specimen strength measured by means of split Hopkinson bar devices and by the output pulse analysis as into the Kolsky-bar solution for metal plasticity [28]. The application of DIF to material models has been the state of the art to model dynamic response of quasi-brittle materials for decades, and it (still) represents its core. A wider experimental approach demonstrated how the material failure limit varies with the specimen/structure size [29], and it was argued that it was the effect of the modification in the crack tip equilibrium due to micro-inertia or other effects [30]. The dynamic strength dependency on specimen size and geometry opens up an important debate regarding the fundamental tests that should be used to characterise the material response, as it strikes directly with the principle of material objectivity for the whole classical concrete material models.

## 3. Materials and Methods

### 3.1. Materials, Specimens and Tests

**Figure 1:**
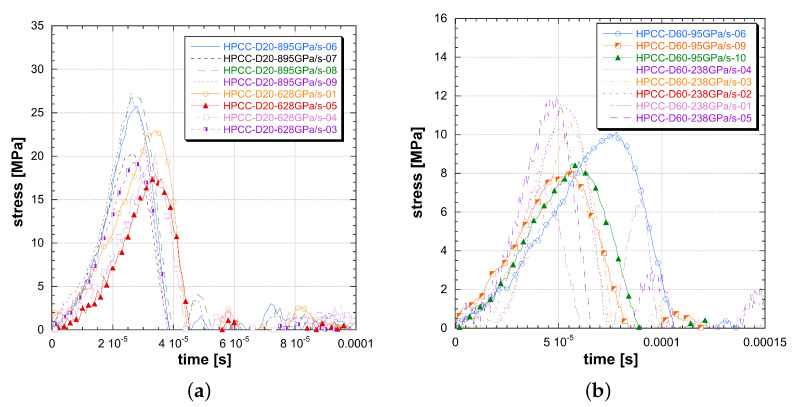
Experimental results tensile stress versus time for the two sizes of ultra-high-performance concretes (UHPCs): (**a**) 20 mm; (**b**) 60 mm.

The extensive experimental program was performed by means of the modified Hopkinson bar apparatus (MHB) based over the original idea of Albertini and Montagnani [31]. More information on the experimental facilities can be found in [32,33]. Out of the whole project data, here, only a group of tests are presented. They were obtained with two MHB tensile configurations (see [19]) having two diameter of 20 mm and 60 mm (d60 and d20), and using cylindrical pre-notched ultra-high-performance concrete (UHPC) specimens subject to tensile loading. The choice to resort to direct tensile stress test allows to study the dynamic response getting rid of the unpredictable perturbations caused by the specimen/bar contacts affecting both indirect tensile and compressive tests. Noise reduction, accuracy and high repeatability are the most appreciated features of the MHB apparatus. Figure 1 shows the experimental results of MHB output pulse applying a tensile load on UHPC pre-notched cylindrical specimens with 20 to 60 mm diameter. In order to compare the experimental outputs, the dynamic loading conditions are here defined by the loading rate. A significant variation in the UHPC material dynamic response was observed. A two times factor both in pulse duration and in failure stress amplitude was evident comparing the two specimens outputs (Figure 1) for a range of dynamic load rate variation from 238 GPa/s to 1 TPa/s.

### 3.2. DAMP Material Model: The Stress–Damage Propagation Interaction

A specimen can be considered as the most simple structure to study the material response; it gives a measure of the material strength which is at the base of every structural calculation, design and evaluation approach. The overall description of the specimen is experimentally captured by average response. The specimen size represents a spatial and geometrical limit beyond which the engineer rarely investigates. M72R3 and RHT material models take the average stress as a model input parameter, neglecting the material–specimen interactions which originates the apparent specimen strength. This clearly represents a limitation to be overcome. In the quasi-static regime, the material–specimen interactions are due to the stress intensity factor for heterogeneous and pre-cracked materials. Instead, in the fast dynamic, there are other phenomena acting inside the specimen volume which create stress and strength dependency to the specimen geometry. The first one is the time- and geometry-dependent propagation of the material modifications under loading (damage and fracture). Those include the shear band propagation velocity for plastic materials and the damage propagation or crack propagation speed for quasi-brittle materials [34]. Crack or damage propagation as well as plasticity propagation are physical transformation induced by stress waves. Those transformations do not occur instantaneously, but they act with a finite speed, observed experimentally and extensively describeded in literature and theoretically justified [35,36].

**Figure 2:**
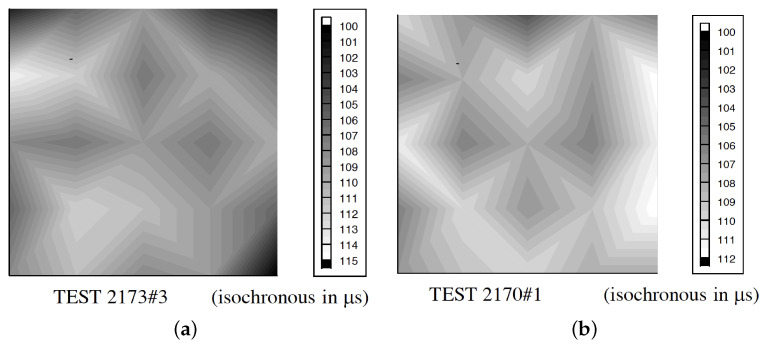
Crack propagation measured by means of the Hopkinson bar bundle device [41] on concrete specimen (cube 200 mm side) @10 s${}^{-1}$: (**a**) dried; (**b**) 50% RH.

The presence of a progressive fracture propagating with finite speed has been experimentally highlighted by several works [37,38]. By a classical overlook, the fracture propagation is consequent to the loads on structure, while the emerging structural strength is due to material strength limit in combination with the structure geometry [39]. In this overlook, the fracture speed is not a relevant parameter for structural engineering. Fracture speed is now focused in its influence to the emerging of the failure limit for specimens and structures. Albertini et al. [18,40,41,42,43,44] showed how, during the specimen dynamic loading up to collapse, the fracture propagates starting from multiple initialisation loci up to the section saturation; this is a core phenomenon composing the final structural strength (Figure 2). This process justifies the sum of the resistance of each subsection which provides the final dynamic response of the structure or specimen. During the dynamic loading, the resistance of each subsection is influenced to the fracture propagation because the propagation of the input load to the measure gauge is possible for the time in which the subsection is undamaged or partially damaged. In this process, the damage propagation speed is the physical parameter fundamental to create the material dynamic response. This new approach is described for structural engineer purposes.

Those last physical observations trigger the idea to describe the material dynamic response by means of the damage propagation speed instead of DIF or other stress characteristics. Based on that, the new material model named DAMP (damage propagation material model) will be here presented.

**Figure 3:**
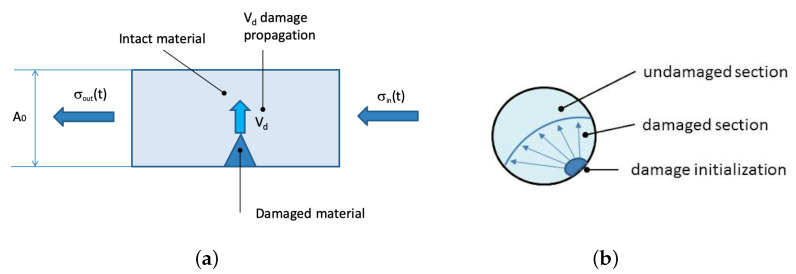
Damage propagation: (**a**) on specimen during a dynamic test; (**b**) on the cross-section.

The case of an SHB apparatus loading a quasi-brittle specimen with a dynamic tensile stress wave is used to describe the DAMP material model idea (Figure 3). The input stress $\sigma_{inp}\left(t\right)$ is the experimental loading wave generated by the SHB apparatus by storing elastic energy into the pretensed bar which is suddenly released [41].

As long as the input wave travels into the input bar, the stress remains into the elastic regime for the bar material. Then, the input wave is transmitted from the input bar to the specimen where it progressively increases the stress over the specimen fracture limit $\sigma_{0}$. The fracture starts at an initialisation point accordingly to the presence of defects or pre-existing cracks. As the fracture starts, it propagates with its speed $V_{d}$ in multiple directions according to the fracture planes and the local stress tensor. Both $V_{d}$ and $\sigma_{0}$ are DAMP material properties associated to the material only.

The crack and the process zone surrounding the crack tip start to propagate through the specimen cross-section (Figure 3b) until they widely extend over it when complete fracture occurs. The propagation of the damage is not instantaneous, but it takes some time depending on its propagation velocity and on the specimen size and geometry. While this occurs, the input wave interacts with the damaged and undamaged parts. In the tensile case, the damaged part of the specimen consists of a set of opened cracks. When the loading wave faces the opened crack, it rebounds back, as the stress transmission to the output bar is not physically possible. Within the intact specimen part, the stress pulse can continue travelling through the output bar because of material continuity, contributing to create the output pulse. This interaction deeply influences the output stress. The output pulse became a quantity related to the material properties and to the geometry of the specimen. Given the nature of the interaction, it is important to split the experimental output pulse into material and geometrical quantities. This is made using the following material model equations.

The instantaneously damaged specimen area $A_{d}$ is calculated by the damage propagation velocity $V_{d}$ and the specimen geometrical characteristics, e.g., diameter $D_{sp}$ or cross-section of the bar $A_{b}$. This results into a symbolic time-dependent function. The more simple damaged area calculation refers to a constant damage propagation speed and a single initialisation point. By the knowledge of the input stress, the specimen characteristics and the two DAMP material constants, the explicit time integration of Equation (8) allows to define the damaged and undamaged area at each time. The wave interaction Equations (9) and (10) allow to transformed the input wave into the test output stress (material apparent strength) and reflected pulse, respectively.

The explicit solution of the whole equation set allows to obtain the specimen fracture time, the output stress wave intensity and amplitude transmitted to the SHB output bar and the reflected pulse. The complete specimen–bar interaction is calculated, resulting in time-, specimen-, material- and input-wave-coupled equations modelling the physical interactions occurring into the quasi-brittle specimen.

(8) $$A_{d}\left(t\right)=g\left(\sigma_{0},V_{d},D_{sp},t\right)$$

(9) $$\sigma_{out}\left(t\right)=\sigma_{inp}\left(t\right)\frac{A_{b}-A_{d}\left(t\right)}{A_{b}}$$

(10) $$\sigma_{r}\left(t\right)=\sigma_{inp}\left(t\right)\frac{A_{d}\left(t\right)}{A_{b}}$$

Unlike the classical material model, the specimen geometry takes an explicit role in shaping the emerging stress pulse. In the DAMP model, the corresponding stress requires a time integration to be solved while respecting geometrical boundary conditions. This has been achieved by implementing the whole SHB test simulation in MATLAB, and in LsDyna finite element code.

### 3.3. Model Application: Constant Loading-Rate Response

From the DAMP equation set, the material model analytic response has been developed in the case of constant velocity of damage propagation and loading with a constant loading rate. The material is loaded with a linear stress in time according to the following equation:

(11) $$\sigma \left(t\right)=\dot{p}\cdot t$$

where $\dot{p}$ is the loading rate (MPa/s) and *t* is the time (s) at which the pulse reaches the notched cross section of the specimen. The initialisation of damage occurs at the activated defects $N_{i}$ which depends on the material characteristic imperfections (const) and on the stress level (σ).

(12) $$N_{i}\left(t\right)=N_{i}\left[\sigma (t),cost\right]$$

In the simplest case, the material model presents one activation once the true critical stress $\sigma_{0}$ is reached at the specimen notch. The damage initialisation requires an average stress over the specimen base $\sigma_{critic-spc}$:

(13) $$\sigma_{critic-spc}=\frac{\sigma_{0}}{k_{i}}$$

which is reached at the time $t_{critic}$ at which the damage propagation starts.

(14) $$t_{critic}=\frac{\sigma_{0}}{\dot{p}\cdot k_{i}}$$

Until this condition is reached, the stress rises with a linear ramp. After $t_{critic}$, the specimen’s apparent strength is conditioned by damage. Here, a new time variable is considered representing the physical time after the damage propagation.

(15) $$t_{2}=t-t_{critic}$$

The apparent material strength $\sigma_{d}$ is obtained as the damaged $A_{d}$ and undamaged $A_{0}$ area contribute to the averaged force over the specimen:

(16) $$\sigma_{d}\left(t\right)=\dot{p}\cdot t\cdot \frac{A_{0}-A_{d}\left(t_{2}\right)}{A_{0}}$$

The damaged area is calculated with a kinematic propagation scheme. In the case of damage propagation starting from the notched side of the specimen, the damaged area is calculated as follow:

(17) $$A_{d}\left(t\right)=\frac{\pi }{2}\cdot N_{i}\cdot {V_{d}}^{2}\cdot {t_{2}}^{2}$$

(18) $$A_{d}\left(t\right)\leq A_{0}$$

The combination of the previous formula leads to a dynamic stress which is a function of time and material/specimen constants:

(19) $$\sigma_{d}=\dot{p}\cdot t\cdot \frac{A_{0}-\frac{\pi }{2}\cdot N_{i}\cdot {V_{d}}^{2}\cdot {\left(t-t_{critic}\right)}^{2}}{A_{0}}$$

for

(20) $$t>t_{critic}$$

That is a cubic function of time *t*.

In the previous equation, the maximum of the dynamic stress is reached at time $t_{m}$. It holds:

(21) $$t_{m}=2\cdot t_{critic}$$

The specimen maximum apparent strength or dynamic strength in the SHB tensile case is given by:

(22) $$\sigma_{m}=2\cdot \dot{p}\cdot t_{critic}-\frac{\dot{p}\cdot \pi \cdot N_{i}\cdot {V_{d}}^{2}\cdot {t_{critic}}^{3}}{A_{0}}$$

or, using the physical variables of the test:

(23) $$\sigma_{m}=2\frac{\sigma_{0}}{k_{i}}-\frac{\pi \cdot N_{i}\cdot {V_{d}}^{2}\cdot {\sigma_{0}}^{3}}{A_{0}{k_{i}}^{2}\dot{p}}$$

The dependence of the maximum dynamic strength on the test conditions and on the material properties is evident.

### 3.4. Structural Solution Comparison between DAMP and Classical Models

**Figure 4:**
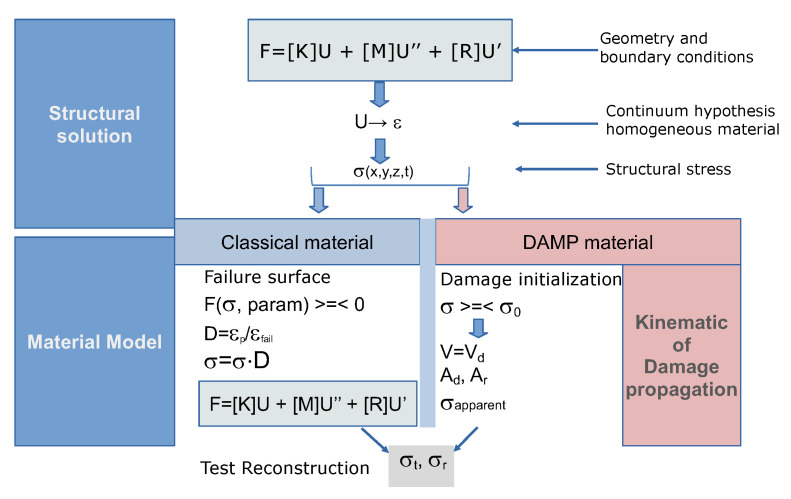
Equation flow to compute structural solution with classical material model and damage propagation (DAMP) material model.

Classical material model calibration as well as its structural use requires the solution of the specimen/structure equilibrium equations. In the following, a symbolic flow allowing to obtain the structural MHB test response starting from the input loads and the constitutive material equations is presented. This is valid for a generic structure by applying the finite element method which automatically provides dynamic equilibrium equations having as input the material, geometry and loads. This general result is resumed in Figure 4 using the symbol of the force vector *F*, stiffness matrix *K*, nodal displacement *U*, mass matrix *M*, nodal acceleration *U*^″^, damp matrix *R* and velocities *U*^′^. By the continuity equations, the strain is calculated starting from the displacements. For an intact elastic material, the stress σ can be calculated at each structure coordinate (*x*, *y*, *z*) at each time *t*.

In case of classical material model, using the previous commented equation set, the final solution of the transmitted and reflected MHB pulse $\sigma_{t}$ and $\sigma_{r}$ can be calculated.

The structural solution procedure obtained with DAMP and the classical model is presented here. The procedure can be applied to any structural case. When the input stress wave invests the specimen, this is generally unloaded or subject to elastic deformations. The dynamic equilibrium equations are applied and solved up to the point at which the failure limit is reached. The use of the fracture initialisation criteria of the DAMP model allows to verify if the material undergoes fracture. Once the failure starts, the material changes its characteristics. The classical model requires computation of a new equation, including the material nonlinearity based on the damage computation. The new equation is written considering the entire specimen volume which undergoes plasticity instantaneously (MHB test case). The main difference between the DAMP model and the classical one consists into the calculation of the instantaneous damage propagation volume, activated by the velocity of damage propagation. By the actual damaged cross section, the equilibrium equation is written considering the undamaged specimen part which responds according to the undamaged material model. Equilibrium allows to obtain the transmitted and reflected MHB pulse stresses $\sigma_{t}$ and $\sigma_{r}$.

Now, having at disposal the gauge test data, the reverse simulation of the test is performed using the chosen constitutive equation. The output pulse is tuned to the experimental output pulse by modifying the material parameters at the best. The simulation obtained by using RHT, M72R3 and DAMP material model are compared.

## 4. Results

Once the material constitutive equation is defined, the material models ability to interpolate simultaneously multiple dynamic tests (see Figure 1) will be verified. Thus, the numerical reconstruction of the MHB test is made as follows: (1) the experimental input stress is assigned to the specimen modelled with the constitutive equations of the material models; (2) the output signal is numerically reconstructed; (3) the experimental output is compared with the one previously computed. In such a way, the best fitting material parameters can be identified.

**Figure 5:**
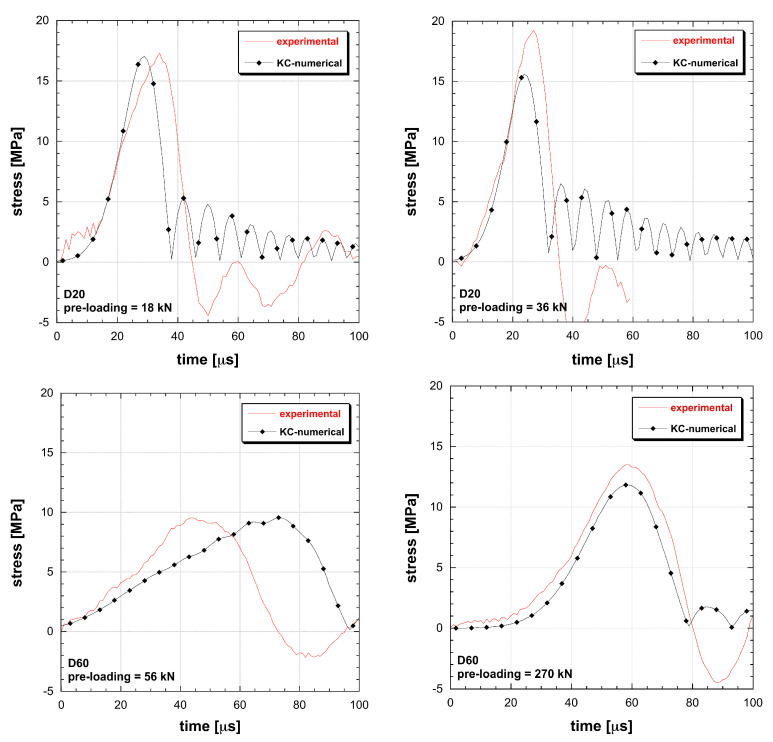
Comparison between experimental and simulated behaviour of UHPC in tension at different loading rate and size (M72R3 (K&C) material model).

**Figure 6:**
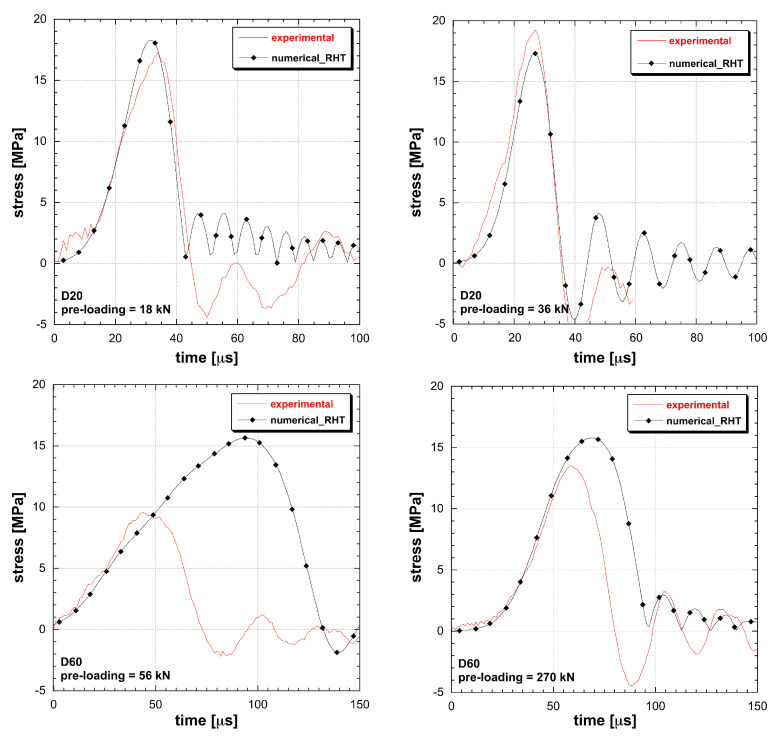
Comparison between experimental and simulated behaviour of UHPC in tension at different loading rate and size (RHT material model).

**Figure 7:**
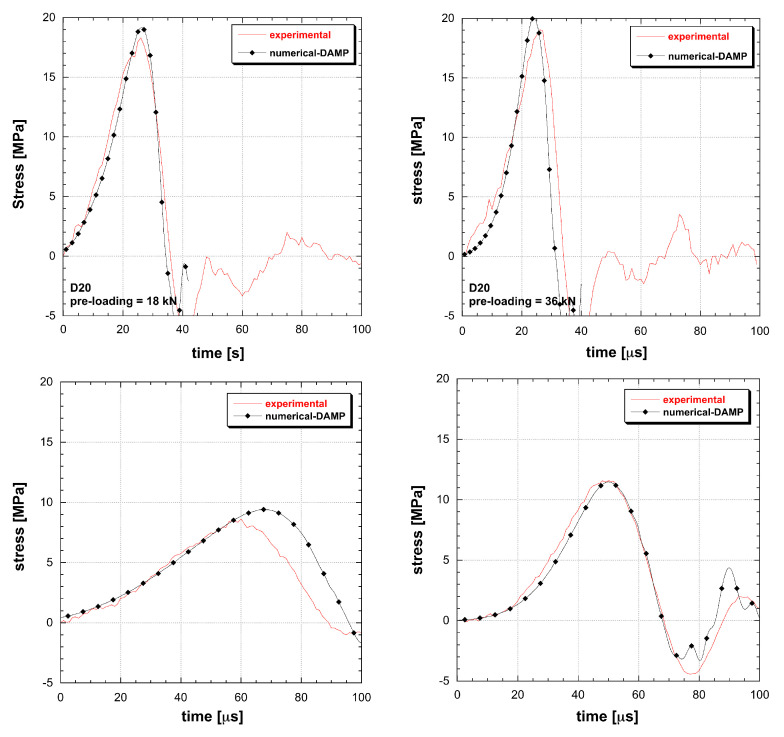
Comparison between experimental and simulated behaviour of UHPC in tension at different loading rate and size (DAMP material model).

The material models based on the DIF function are not able to interpolate multiple tests. A conflict in amplitude and duration occurs: the more tests are added, the more interpolation constraints emerge (Figure 5 and Figure 6). The impossibility to reproduce the dynamic tensile response leads to the introduction of unknown final errors into numerical applications as well as to a nonphysical damage calculation. The eventual introduction of a best-fit approach for material identification does not result into more reliable performances but just spreads the errors within the whole test cases. When dealing with RHT and M72R3 numerical test reconstructions, it emerges how two tests are the limit of these material models to obtain a good interpolation. Once the user applies the material identification by using a limited number of tests, a couple of test conditions is correctly interpolated, while the material remains unable to adapt to different geometry or dynamic loads. So that, the DIF resulting from a specimen set is dependent on the particular geometrical solution of the test, in contrast with the principle of material objectivity. Instead the DAMP material model is able to fit all the experiments considered, overcoming the contradiction present in classical models (Figure 7).

## 5. Discussion

**Figure 8:**
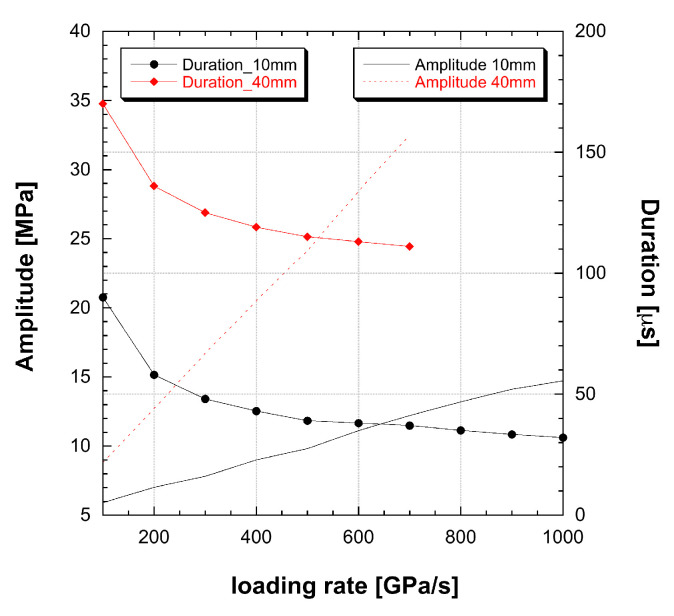
Dynamic strength and amplitude obtained by DAMP model on D10 and D40 specimens.

The structural outputs calculated by the use of different material models are now parametrised to specimen size variations and loading-rate. This approach generalises the results observed reconstructing the experimental data. By adopting RHT and M72R3 material models, or any other material model based on stress–strain relation and DIF dynamic characterisation, any specimen dynamic strength output is represented by the DIF itself. That is by imposing the same loading rate to specimens of increasing size, an identical dynamic strength is obtained. Within the DAMP material model, the specimen size plays a direct role into the constitutive equations by means of the damage propagation. The DAMP interaction equations are analytically solved in case of loads increasing with time according to the loading rate $\dot{p}$. Considering a circular propagation of the damage, the set of Equations (18)–(20) provides the output strength with a time-dependent amplitude (Equation (24)) [34]:

(24) $$\sigma_{d}\left(t\right)=\dot{p}\cdot t\cdot \left[1-\frac{\pi \cdot N_{i}\cdot {V_{d}}^{2}\cdot {\left(t-t_{critic}\right)}^{2}}{2\cdot A_{0}}\right]$$

where $N_{i}$ is the number of crack initialisation and $A_{d}$ is the specimen area. By applying the DAMP model (Equation (24)) to specimen size 10 and 40 mm, considering experimental loading conditions loading rate from 100 GPa/s to 1 TPa/s, the output pulse amplitude and duration have been calculated and depicted in graph Figure 8.

The DAMP dynamic strength calculated with diameter 10 or 40 mm specimens represents a single pseudo-plastic material model set belonging to the classical theory, as each curve can be obtained with a classical material model using a different DIF curve. In general, each time the specimen changes size, a modification into the DIF is obtained as the specimen dynamic strength changes.

Because the output pulse depends on geometrical parameters, a brittle material can generate a family of different output pulse depending on the test boundary, geometrical and loading conditions. This phenomenon is here named multiple pseudo-plastic appearance of brittle materials. The identification of the output pulse in material stress–strain properties incorporates geometrical properties into the DIF, leading to material models intrinsically not objective and not suitable to be applied to different type of structures.

**Figure 9:**
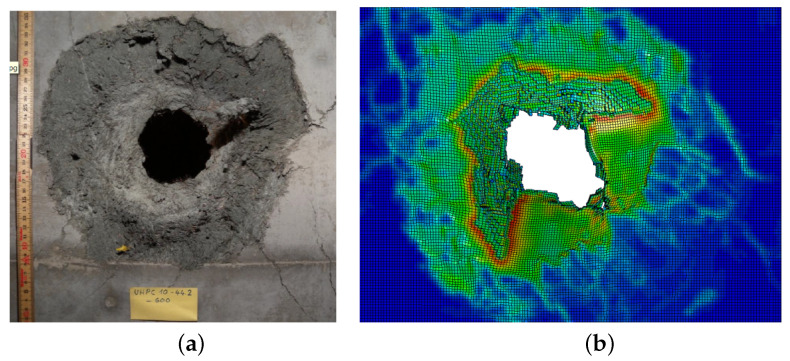
Experimental (**a**) and numerical reconstruction (**b**) of structural test using the finite element method and the DAMP material model (courtesy of armasuisse S+T).

By the DAMP formulation, the use of DIF can be avoided and the dynamic strength is a structural result obtained by the material application to the structure. This material model has proved to be closer to the material model objectivity principle increasing then the calculation accuracy in the framework presented data and, more in general, providing a promising approach to the analysis of structures subject to blast tests in the frame of the activities developed in cooperation with armasuisse S+T (Figure 9).

## 6. Concluding Remarks

Structural engineers need in everyday practice reliable material models and properties to develop widely and trustworthy structural assessment. The robustness of the structural calculation is the main attribute to save money in real scale tests and to ensure the safety requirements. The need to improve the structural reliability lead to reconsider material model equations. This has been achieved using an experimental parametric approach and numerical reconstruction of the results using different material models. Considering the fracture mechanic results, the new material model DAMP has been proposed. Relevant differences with respect to classical models in the ability to interpolate multiple dynamic tests were demonstrated. The classical choice of the material properties is based on stress and strains and dynamic strength. This choice simply fits the engineering needs but creates material models not objectives. The mathematical upgrade of material models does not solve the objectivity problems present, leading to a lack of reliability in a wide range of applications. Those material models can interpolate few dynamic tests at a time, while they prove to be inaccurate when considering more tests, showing strong limitations in case of specimen or structure size variations.

Using the damage propagation as the primary material model variable into the DAMP material model, a wide range of dynamic tests were accurately reconstructed including specimen size variations. It has been shown how the real process of quasi-brittle fracture is closer to the DAMP equation set than to the classical model and that, because of the stress wave–fracture interaction, the dynamic strength is a quantity sensitive to specimen size. This interaction has been neglected in classical material models and is the primary source of their inaccuracy. Through a parametric approach, it has been shown how the fracture process or a single DAMP parameter generates a multitude of DIF curves corresponding to several material models set in a classical overlook. The multiple pseudo-plastic appearance has been defined as the emerging of multiple classical material model parameters by an identical constructive material because of its brittle fracture nature. This effect causes important restrictions in predictiveness capability of classical brittle material models.
